# Incretin hormone responses to carbohydrate and protein/fat are preserved in adults with sulfonylurea‐treated *KCNJ11* neonatal diabetes

**DOI:** 10.1111/jdi.14071

**Published:** 2023-08-21

**Authors:** Pamela Bowman, Kashyap A Patel, Timothy J McDonald, Jens J Holst, Bolette Hartmann, Maria Leveridge, Beverley M Shields, Suzie Hammersley, Steve R Spaull, Bridget A Knight, Sarah E Flanagan, Maggie H Shepherd, Rob C Andrews, Andrew T Hattersley

**Affiliations:** ^1^ University of Exeter Medical School Exeter UK; ^2^ Exeter NIHR Clinical Research Facility Exeter UK; ^3^ NIHR Exeter Biomedical Research Centre (BRC) Exeter UK; ^4^ Royal Devon University Healthcare NHS Foundation Trust Exeter UK; ^5^ Faculty of Health and Medical Sciences University of Copenhagen Kobenhavn Denmark

**Keywords:** GIP, GLP‐1, incretin hormones, Neonatal diabetes, sulfonylurea

## Abstract

The incretin hormones glucagon‐like peptide‐1 (GLP‐1) and glucose‐dependent insulinotropic polypeptide (GIP), are thought to be the main drivers of insulin secretion in individuals with sulfonylurea (SU)‐treated *KCNJ11* permanent neonatal diabetes. The aim of this study was to assess for the first time the incretin hormone response to carbohydrate and protein/fat in adults with sulfonylurea‐treated *KCNJ11* permanent neonatal diabetes compared with that of controls without diabetes. Participants were given a breakfast high in carbohydrate and an isocaloric breakfast high in protein/fat on two different mornings. Incremental area under the curve and total area under the curve (0‐240 minutes) for total GLP‐1 and GIP were compared between groups, using non‐parametric statistical methods. Post‐meal GLP‐1 and GIP secretion were similar in cases and controls, suggesting this process is adenosine triphosphate‐sensitive potassium channel‐independent. Future research will investigate whether treatments targeting the incretin pathway are effective in individuals with *KCNJ11* permanent neonatal diabetes who do not have good glycemic control on sulfonylurea alone.

## Graphical abstract

Insulin secretion in individuals with sulfonylurea‐treated *KCNJ11* permanent neonatal diabetes is thought to be dependent on non‐ adenosine triphosphate‐sensitive potassium channel pathways; for example, incretin hormones. We assessed incretin hormone secretion after different foods in individuals with sulfonylurea‐treated *KCNJ11* permanent neonatal diabetes, in comparison with people without diabetes. Patterns of glucose‐dependent insulinotropic polypeptide and glucagon‐like peptide‐1 secretion after protein/fat and carbohydrate meals were similar in cases and controls. An intact incretin pathway might provide a therapeutic target for individuals with sulfonylurea‐treated *KCNJ11* permanent neonatal diabetes who require adjunctive therapy to optimize glycemic control.

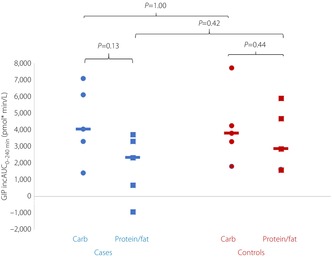

## INTRODUCTION

Heterozygous activating mutations in the *KCNJ11* gene cause permanent neonatal diabetes (PNDM) by rendering the Kir6.2 subunit of the pancreatic adenosine triphosphate (ATP)‐sensitive (K_ATP_) channel insensitive to ATP^1^. The molecular defect can be overcome by sulfonylurea (SU) drugs, which still bind mutant K_ATP_ channels and facilitate endogenous insulin secretion^2^. Affected individuals can be treated successfully with high doses of SU instead of insulin injections, maintaining excellent glycemic control long term^3^.

Regulation of insulin secretion in SU‐treated *KCNJ11*‐PNDM cannot occur through the classical ATP pathway, and is thought to be driven predominantly by the incretin hormones, glucagon‐like peptide‐1 (GLP‐1) and glucose‐dependent insulinotropic polypeptide (GIP)^2^. We previously described physiological responses to carbohydrate and protein/fat meals in affected adults; insulin secretion was similar after the two meals, despite higher glucose levels after carbohydrate than protein/fat. This pattern was not seen in controls, who matched insulin secretion to the content of the meal^4^.

Understanding the unique physiology of people with SU‐treated *KCNJ11*‐PNDM is important both scientifically and therapeutically. Affected individuals represent human models for the study of non‐K_ATP_‐channel pathways, including those relating to the secretion of GLP‐1 and GIP from L cells and K cells. In addition, drugs targeting incretin pathways might be a potential adjunct to treatment for the minority of patients with *KCNJ11*‐PNDM who do not achieve good glycemic control on SU monotherapy^5^, ^6^. Despite these implications, no studies have assessed the incretin responses to different foods in these patients.

Our aim was to assess the GLP‐1 and GIP responses to carbohydrate and protein/fat meals in adults with *KCNJ11*‐PNDM, and to compare these with adults without diabetes.

## MATERIALS AND METHODS

### Clinical studies

Sample collection took place as part of a previously reported study^4^. Five cases aged >18 years with SU‐treated *KCNJ11*‐PNDM, and five age‐, sex‐ and body mass index‐matched controls without diabetes (Table S1) fasted overnight, then consumed a high carbohydrate meal and an isocaloric high protein/fat meal (in random sequence) on two different mornings. Cases took their prescribed SU medication with each meal. Cases had an additional visit, during which they took SU without food. Blood samples were taken at baseline (−5 and 0 minutes), 15, 30, 45, 60, 90, 120, 150, 180, 210 and 240 min post‐meal, and spun and frozen at −80°C.

Minor deviations from the meal and sampling protocol were previously reported^4^. One case had an increase in prescribed gliclazide between the second and third visits (from 200 to 320 mg daily).

### Biochemical analysis

Plasma concentrations of total GIP and total GLP‐1 were measured with commercially available sandwich enzyme‐linked immunosorbent assay kits (cat no: 10‐1258‐01 and 10‐1278‐01; Mercodia, Uppsala, Sweden). The detection limit for the GIP enzyme‐linked immunosorbent assay is 1.62 pmol/L, and for the GLP‐1 enzyme‐linked immunosorbent assay the detection limit is 0.65 pmol/L.

### Statistical analysis

Data were analyzed in StataSE Version 17 (StataCorp, College Station, TX, USA). Incremental area under the curve (iAUC_0–240 min_); that is, the change from baseline on the day of each visit, and total area under the curve (tAUC_0–240 min_) over 240 min, were calculated for GIP and GLP‐1 after each meal (and without food for cases). Values were compared between cases and controls, and between meals for each group. Non‐parametric statistical methods were used (Wilcoxon signed‐rank test for paired continuous data and Mann–Whitney test for unpaired continuous data [independent samples]). Data are presented as the median (range), unless otherwise stated.

## RESULTS

**Figure 1:**
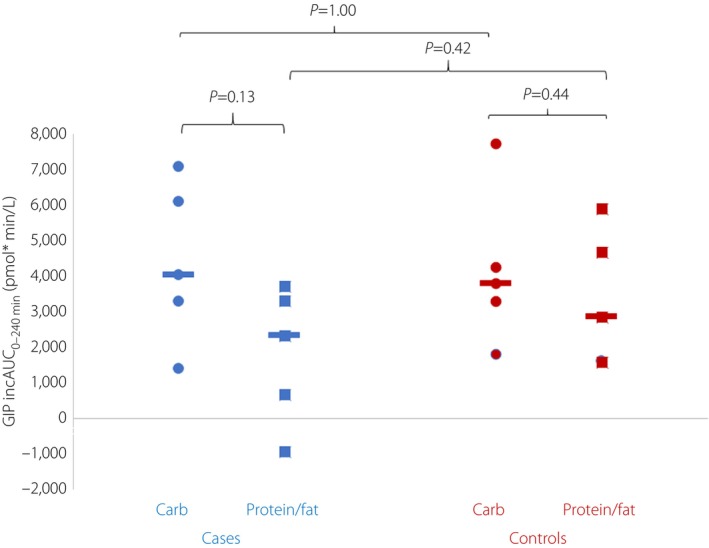
Glucose‐dependent insulinotropic polypeptide (GIP) incremental area under the curve (iAUC_0–240 min_) in *KCNJ11* permanent neonatal diabetes cases (*n* = 5) and controls (*n* = 5) with carbohydrate versus protein/fat meal.

Incremental GIP levels were similar in cases and controls after protein/fat and carbohydrate meals (iAUC_0–240 min_ for GIP after protein/fat 2,319 [−947 to 3,713] vs 2,841 [1,568–5,895] pmol × min/L, *P* = 0.42 and after carbohydrate 4,013 [1,369–7,064] vs 3,787 [1,784–7,712] pmol × min/L, *P* = 1.00), Figure 1 and Figure S1. A similar pattern was observed with total GIP levels (Table S2).

**Figure 2:**
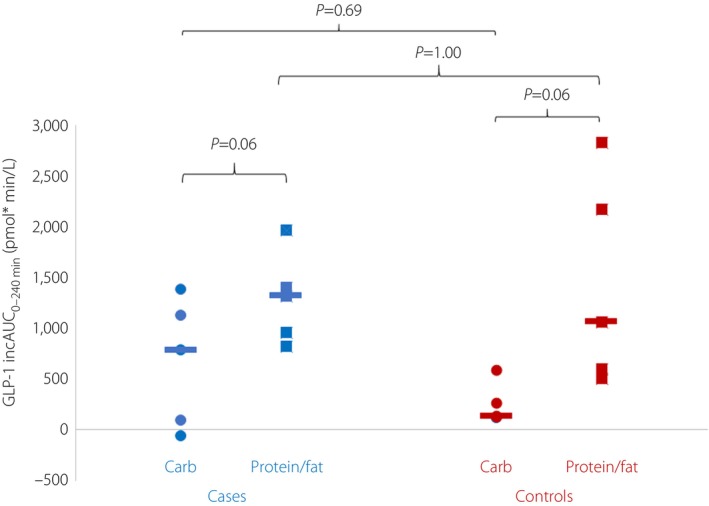
Glucagon‐like peptide‐1 (GLP‐1) incremental area under the curve (iAUC_0–240 min_) in *KCNJ11* permanent neonatal diabetes cases (*n* = 5) and controls (*n* = 5) with carbohydrate versus protein/fat meal.

Incremental GLP‐1 levels were similar in cases and controls after protein/fat and carbohydrate meals (iAUC_0–240 min_ for GLP‐1 after protein/fat 1,313 [822–1,965] vs 1,061 (505–2,822) pmol × min/L, *P* = 1.00 and after carbohydrate 783 [−63 to 1,378] vs 131 [113–585] pmol × min/L, *P* = 0.69; Figure 2 and Figure S2). A similar pattern was observed with total GLP‐1 levels after a protein meal/fat meal; after a carbohydrate meal, total GLP‐1 levels were higher in cases than controls (*P* = 0.03), Table S2.

There was a trend toward more potent stimulation of GLP‐1 secretion by protein/fat than carbohydrate in both cases (iAUC_0–240 min_ 1,313 [822–1,965] vs 783 [−63 to 1,378] pmol × min/L, *P* = 0.06) and controls (iAUC_0–240 min_ 1,061 [505–2,822] vs 131 [113–585] pmol × min/L, *P* = 0.06; Figure 2). In contrast, GIP secretion was similar irrespective of the meal type in cases (iAUC_0–240 min_ 2,319 [−947 to 3,713] pmol × min/L after protein/fat vs 4,013 [1,369–7,064] pmol × min/L after carbohydrate, *P* = 0.13) and controls (iAUC 2,841 [1,568–5,895] pmol × min/L after protein/fat vs 3,787 [1,784–7,712] pmol × min/L, *P* = 0.44; Figure 1).

In cases, incretin hormone secretion was higher after both meals when compared with SU alone (GLP‐1 iAUC_0–240 min_ protein/fat vs no food 1,313 [822–1,965] vs −173 [−703 to 240] pmol × min/L, *P* = 0.06, carbohydrate vs no food 783 [−63 to 1,378] vs −173 [−703 to 240] pmol × min/L, *P* = 0.06, and GIP iAUC_0–240 min_ protein/fat vs no food 2,319 [−947 to 3,713] vs −124 [−1,291 to −86] pmol × min/L, *P* = 0.06, carbohydrate vs no food 4,013 [1,369–7,064] vs −124 [−1,291 to −86] pmol × min/L, *P* = 0.06). Similar patterns were observed with tAUC (Figure S3, Table S2).

## DISCUSSION

We have shown that the incretin response is similar in adults with SU‐treated *KCNJ11*‐PNDM when compared with healthy controls after protein/fat and carbohydrate meals. Incremental GLP‐1 and GIP secretion is of similar magnitude in both groups. In addition, protein/fat results in greater GLP‐1 secretion than carbohydrate, in keeping with previous research^7^.

The preserved incretin response in adults with *KCNJ11*‐PNDM contrasts with the reduced post‐meal GLP‐1 that is often seen in adults with type 2 diabetes^8^. Although GIP secretion can remain near‐normal in individuals with type 2 diabetes, the insulin response to GIP is markedly impaired; in contrast, the insulin response to GLP‐1 is preserved^9^. The adults in our study had lower insulin secretion after a carbohydrate meal despite higher glucose values in comparison with controls^4^, which is most likely a result of impaired ATP sensitivity at the level of the pancreatic K_ATP_ channel. Further research is needed to investigate whether the insulin response to GIP is affected in these individuals.

The present research provides mechanistic insights. Preserved incretin hormone secretion in individuals with mutant K_ATP_ channels suggests that GLP‐1 and GIP secretion from L cells and K cells is independent of K_ATP_ channels, consistent with previous rodent and human studies^2^, ^10^.

The present study also has therapeutic implications. Although long‐term SU monotherapy is effective in the majority of individuals with *KCNJ11*‐PNDM, a proportion of patients require adjunctive medication to optimize glycemic control, including the reintroduction of insulin^3^. Our data support consideration of the use of second‐line treatments that further augment the incretin pathway, consistent with two previous case reports^5^, ^6^. Dual GIP and GLP‐1 receptor agonists might have future therapeutic potential^11^.

However, an important consideration is the possible direct effects of sulfonylureas on incretin hormone secretion or action. Previous studies in perfused rat pancreas suggest that high‐dose sulfonylureas cause dissociation of the insulinotropic effects of GLP‐1 from glucose levels; at 3 mmol/L glucose, tolbutamide alone resulted in increased insulin secretion that was not seen with GLP‐1 alone, whereas administration of both GLP‐1 and tolbutamide augmented insulin secretion much more markedly^12^. More recently, studies in humans with type 2 diabetes have shown that low‐dose gliclazide, which does not stimulate insulin secretion at low glucose levels, augments insulin secretion in response to oral, but not intravenous, glucose^13^. The latter scenario might mimic more closely the mechanism of action of sulfonylureas in the context of *KCNJ11*‐related neonatal diabetes, as although high doses are used, they are thought to act largely permissively in allowing β‐cells to respond to incretins, rather than being directly stimulatory^2^. Our data show very limited effects of sulfonylureas alone on insulin^4^ or incretin secretion, but a drop in glucose was still observed in the context of sulfonylurea with no food^4^. Further research is needed to investigate the mechanism of action of sulfonylureas and interactions with drugs that augment the incretin pathway in this physiologically unique group of patients.

The present study had limitations. First, the sample size was small. However, to our knowledge, this is the only study measuring GIP and GLP‐1 levels in individuals with *KCNJ11*‐PNDM, in response to different nutrients. Second, the study was limited to individuals aged >18 years. More research is needed to investigate incretin hormone secretion in children with *KCNJ11*‐PNDM. An additional limitation is that there was no comparison with a control group on sulfonylurea treatment; for example, adults with type 2 diabetes. This will be an important area to address in future research studies.

In conclusion, we have shown that incretin hormone responses are preserved in adults with SU‐treated *KCNJ11*‐PNDM after carbohydrate and protein/fat meals, in comparison with healthy controls. This suggests that K_ATP_ channels do not play a role in GIP and GLP‐1 secretion. Future research will establish the utility of GLP‐1 agonists and DPP‐IV inhibitors as adjunctive treatments in individuals with *KCNJ11*‐PNDM who have suboptimal glycemic control on SU monotherapy.

## DISCLOSURE

The authors declare no conflict of interest.

Approval of the research protocol: The study was approved by the South West–Cornwall and Plymouth Research Ethics Committee and the Health Research Authority (REC reference 16/SW/0150).

Informed consent: All participants provided informed consent before taking part.

Registry and the registration no. of the study/trial: Clinicaltials.gov identifier NCT02921906, approved on 3rd October 2016.

Animal studies: N/A.

## Supporting information


**Figure S1** | (a) Median incremental GIP secretion in *KCNJ11* PNDM cases (blue) and controls (red) with carbohydrate (solid line) and protein/fat (dashed line) meal. (b) Mean (SD) incremental GIP secretion in *KCNJ11* PNDM cases.
**Figure S2** | (a) Median incremental GLP‐1 secretion in *KCNJ11* PNDM cases (blue) and controls (red) with carbohydrate (solid line) and protein/fat (dashed line) meal. (b) Mean (SD) incremental GIP secretion in *KCNJ11* PNDM cases.
**Figure S3** | (a) Median GLP‐1 and GIP secretion (absolute values) in *KCNJ11* PNDM cases with carbohydrate (blue), protein/fat (red) and no food/SU only (black). (b) Mean (SD) GLP‐1 and GIP secretion (absolute values) in *KCNJ11* PNDM cases with carbohydrate (blue), protein/fat (red) and no food/SU only (black).
**Table S1** | Clinical characteristics of study participants (at baseline).
**Table S2** | Total area under the curve (tAUC) for GLP‐1 and GIP after different meals in *KCNJ11* cases and healthy controls.
