# Supplementary material for: Incretin hormone responses to carbohydrate and protein/fat are preserved in adults with sulfonylurea‐treated KCNJ11 neonatal diabetes
Source: J Diabetes Investig. 2023 Aug 21;14(12):1378–82. doi: 10.1111/jdi.14071 (PMC10688132; doi:10.1111/jdi.14071)
Supplement: Supplementary file 1 — Figure S1 | (a) Median incremental GIP secretion in KCNJ11 PNDM cases (blue) and controls (red) with carbohydrate (solid line) and protein/fat (dashed line) meal. (b) Mean (SD) incremental GIP secretion in KCNJ11 PNDM cases. Figure S2 | (a) Median incremental GLP‐1 secretion in KCNJ11 PNDM cases (blue) and controls (red) with carbohydrate (solid line) and protein/fat (dashed line) meal. (b) Mean (SD) incremental GIP secretion in KCNJ11 PNDM cases. Figure S3 | (a) Median GLP‐1 and GIP secretion (absolute values) in KCNJ11 PNDM cases with carbohydrate (blue), protein/fat (red) and no food/SU only (black). (b) Mean (SD) GLP‐1 and GIP secretion (absolute values) in KCNJ11 PNDM cases with carbohydrate (blue), protein/fat (red) and no food/SU only (black). Table S1 | Clinical characteristics of study participants (at baseline). Table S2 | Total area under the curve (tAUC) for GLP‐1 and GIP after different meals in KCNJ11 cases and healthy controls. [file JDI-14-1378-s001.pptx]

## Slide 1
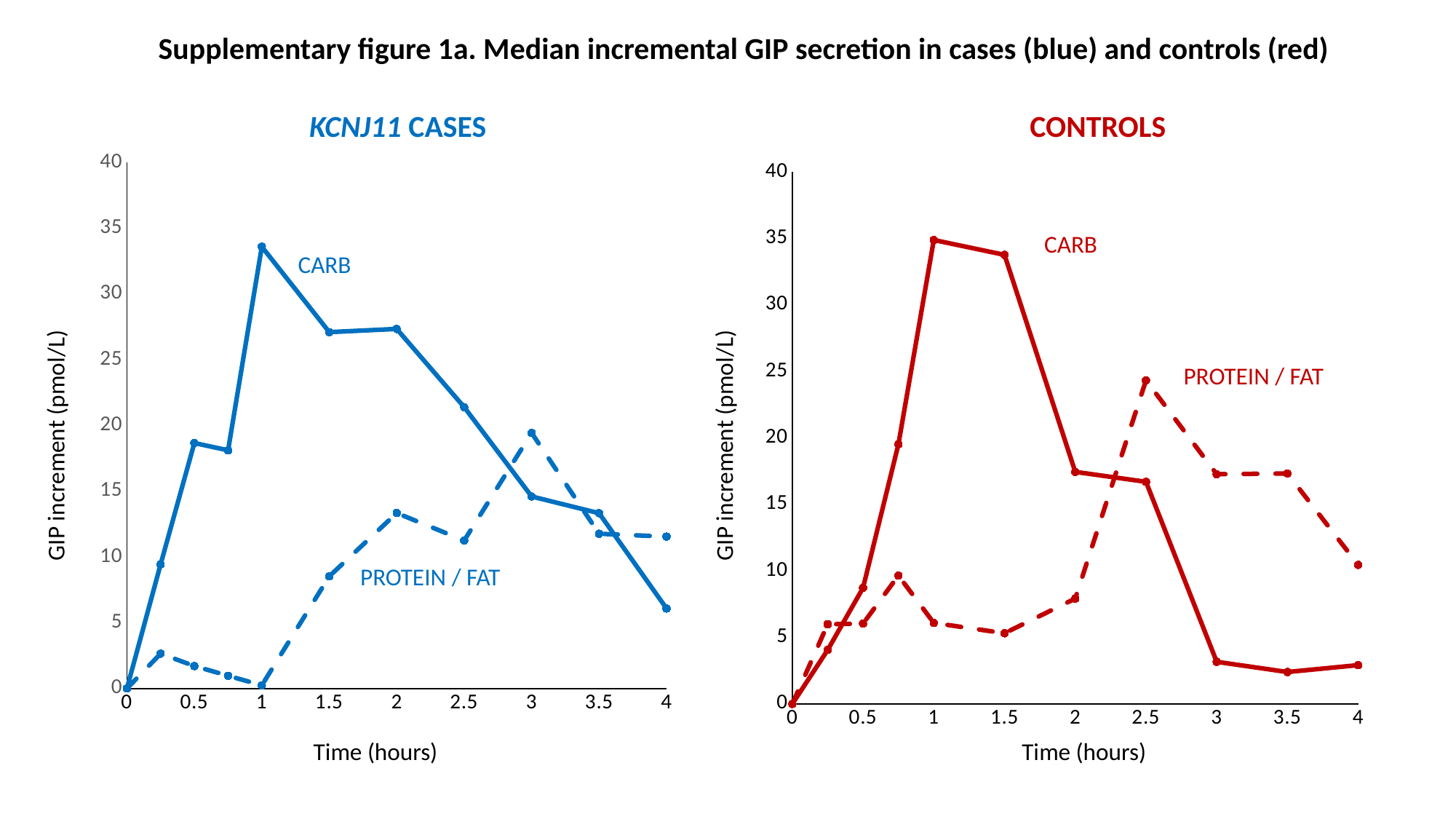

Supplementary figure 1a. Median incremental GIP secretion in cases (blue) and controls (red)
KCNJ11 CASES
CONTROLS
### Chart
| Category | | |
|---|---|---|
### Chart
| Category | | |
|---|---|---|CARB
CARB
PROTEIN / FAT
GIP increment (pmol/L)
GIP increment (pmol/L)
PROTEIN / FAT
Time (hours)
Time (hours)

## Slide 2
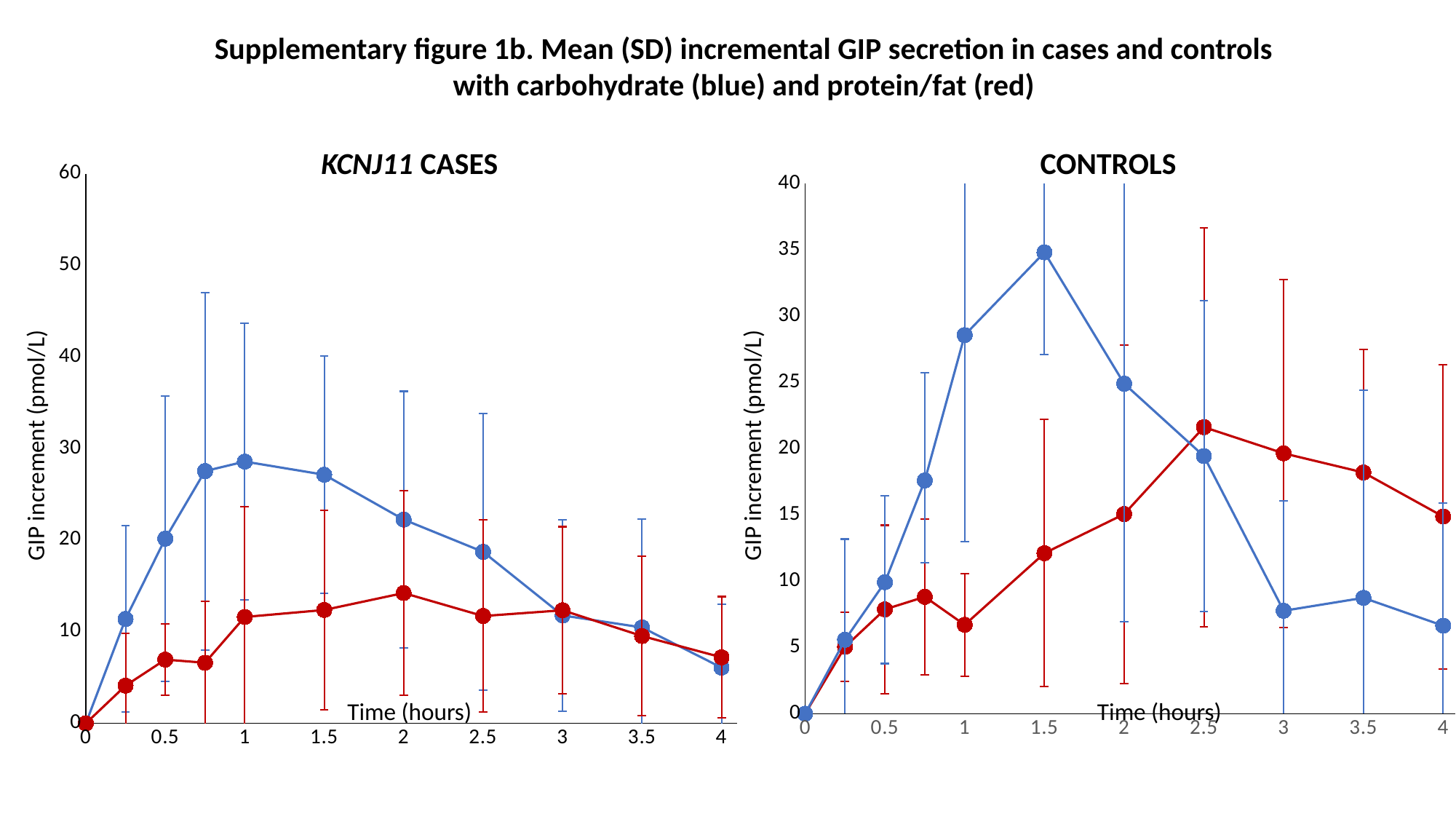

Supplementary figure 1b. Mean (SD) incremental GIP secretion in cases and controls
with carbohydrate (blue) and protein/fat (red)
KCNJ11 CASES
CONTROLS
### Chart
| Category | | |
|---|---|---|
### Chart
| Category | | |
|---|---|---|GIP increment (pmol/L)
GIP increment (pmol/L)
Time (hours)
Time (hours)

## Slide 3
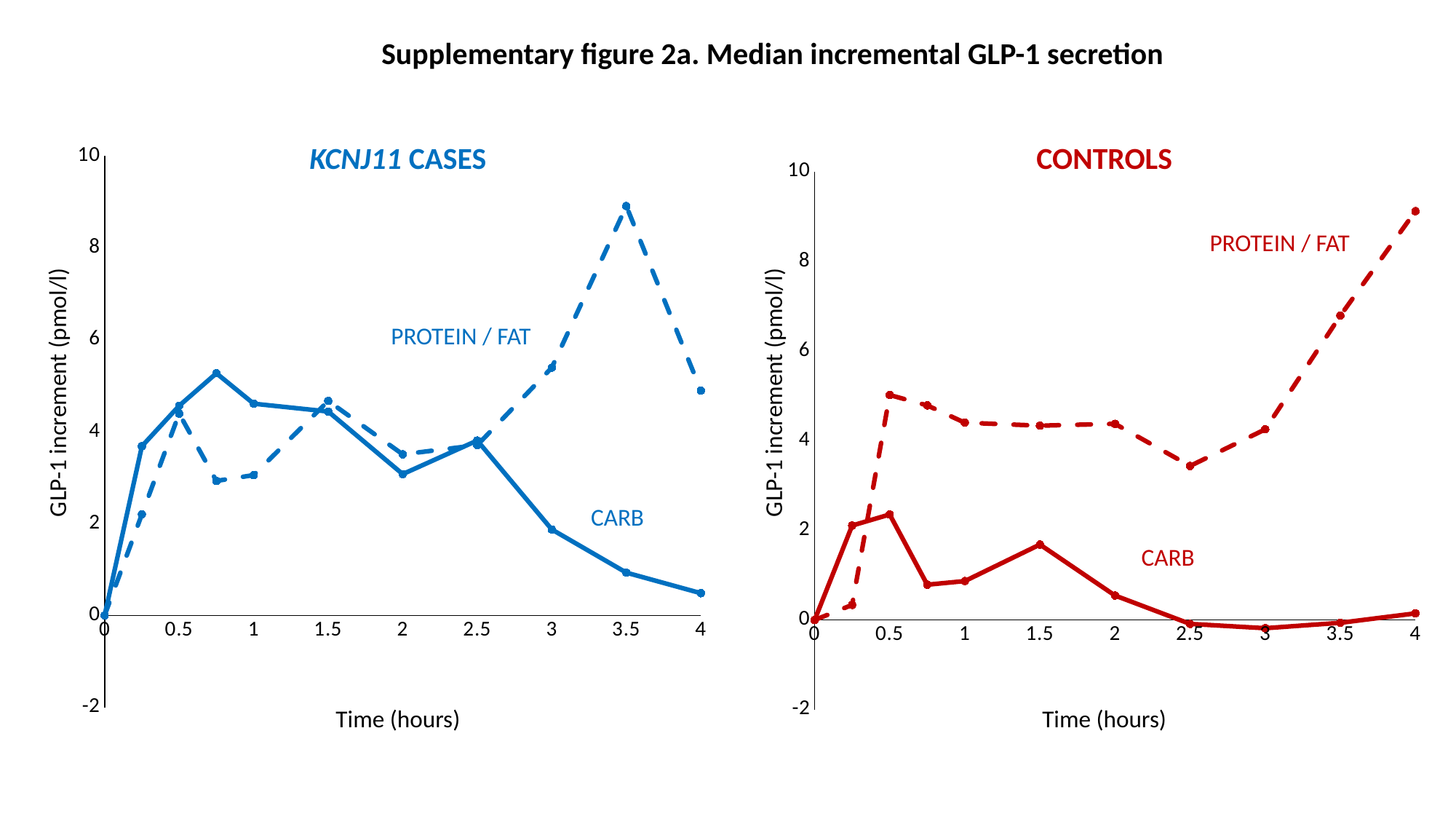

Supplementary figure 2a. Median incremental GLP-1 secretion
### Chart
| Category | | |
|---|---|---|KCNJ11 CASES
CONTROLS
### Chart
| Category | | |
|---|---|---|PROTEIN / FAT
PROTEIN / FAT
GLP-1 increment (pmol/l)
GLP-1 increment (pmol/l)
CARB
CARB
Time (hours)
Time (hours)

## Slide 4
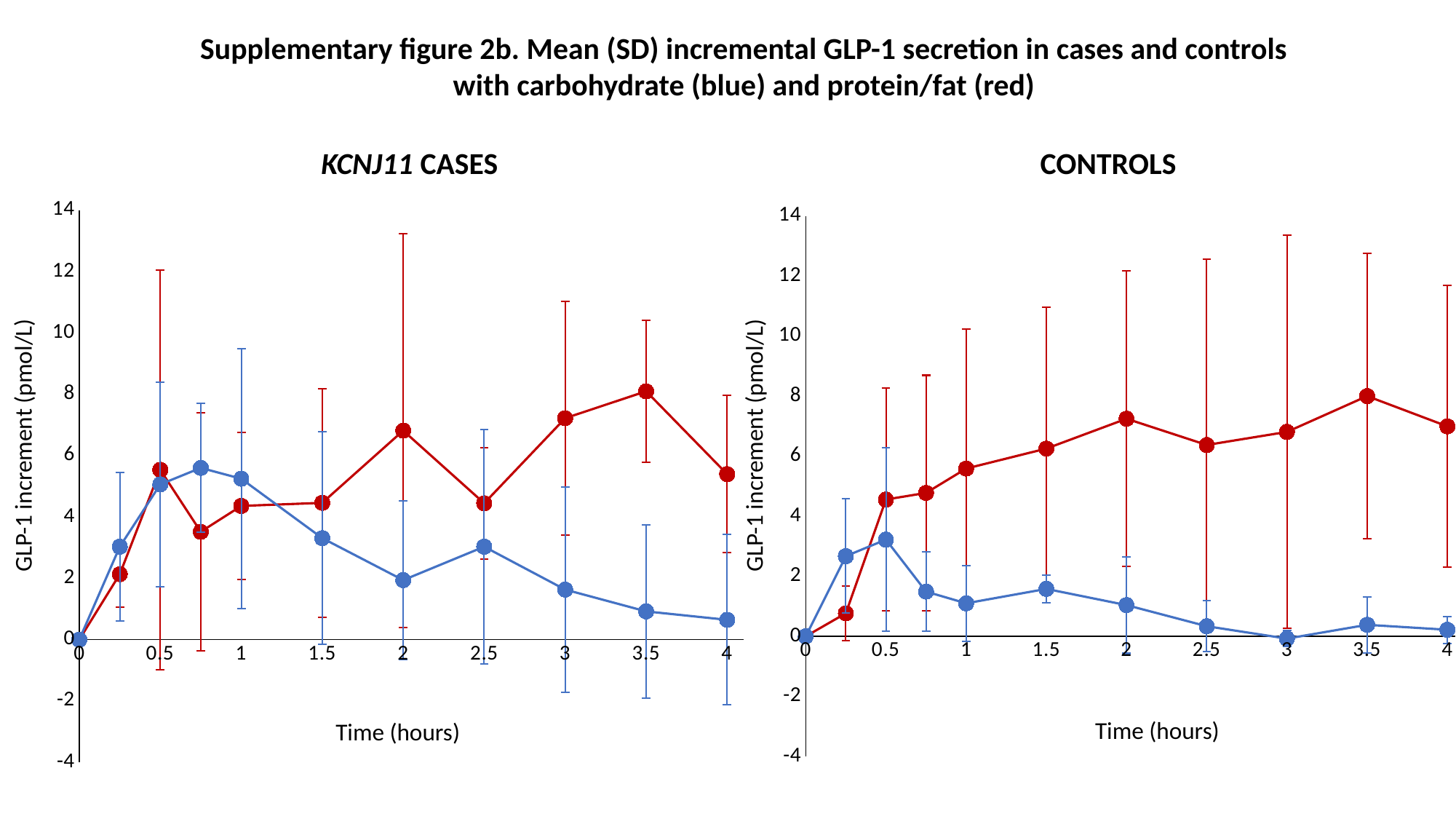

Supplementary figure 2b. Mean (SD) incremental GLP-1 secretion in cases and controls
with carbohydrate (blue) and protein/fat (red)
KCNJ11 CASES
CONTROLS
### Chart
| Category | | |
|---|---|---|
### Chart
| Category | | |
|---|---|---|GLP-1 increment (pmol/L)
GLP-1 increment (pmol/L)
Time (hours)
Time (hours)

## Slide 5
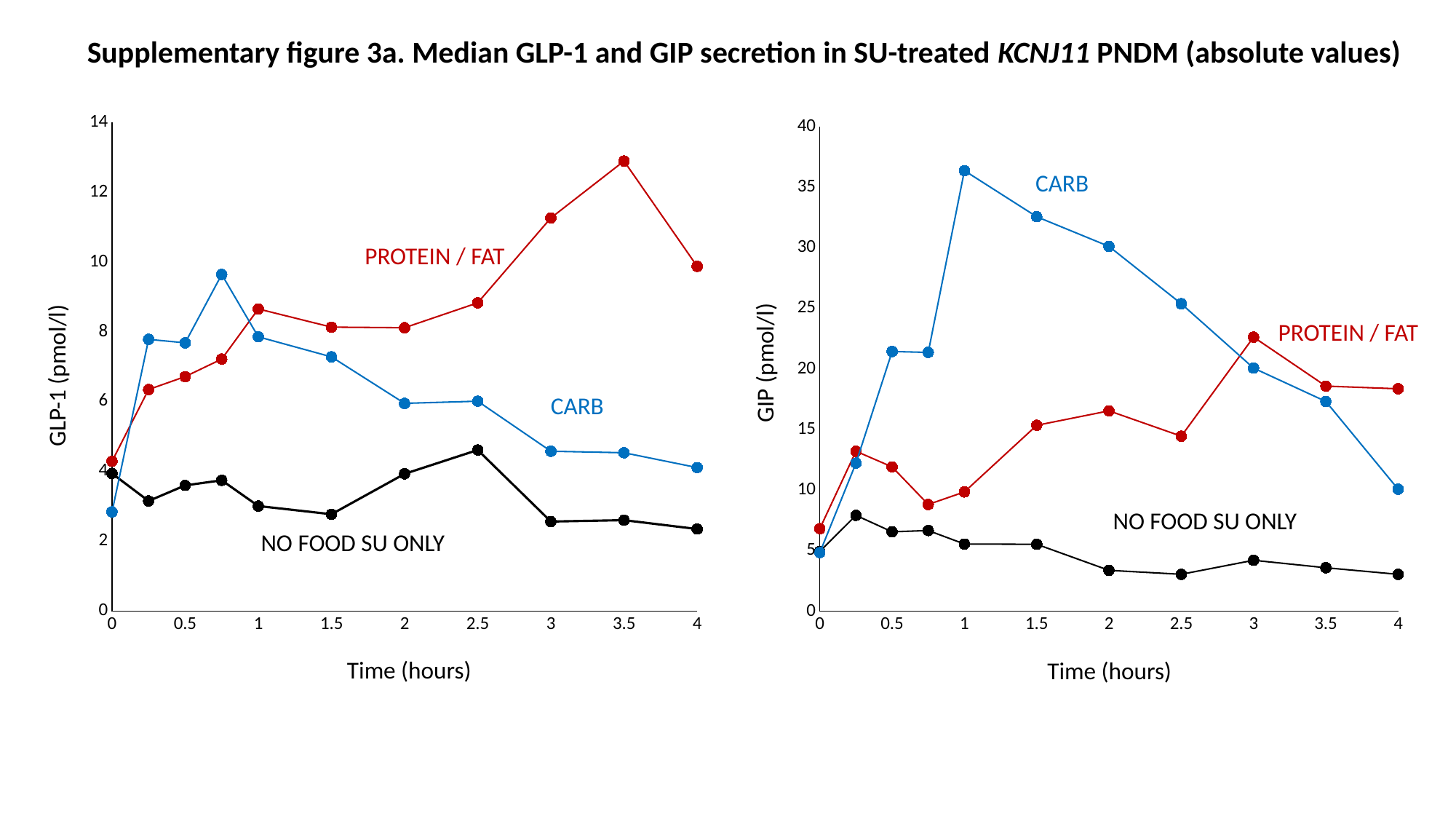

Supplementary figure 3a. Median GLP-1 and GIP secretion in SU-treated KCNJ11 PNDM (absolute values)
### Chart
| Category | | | |
|---|---|---|---|
### Chart
| Category | | | |
|---|---|---|---|CARB
PROTEIN / FAT
PROTEIN / FAT
GIP (pmol/l)
GLP-1 (pmol/l)
CARB
NO FOOD SU ONLY
NO FOOD SU ONLY
Time (hours)
Time (hours)

## Slide 6
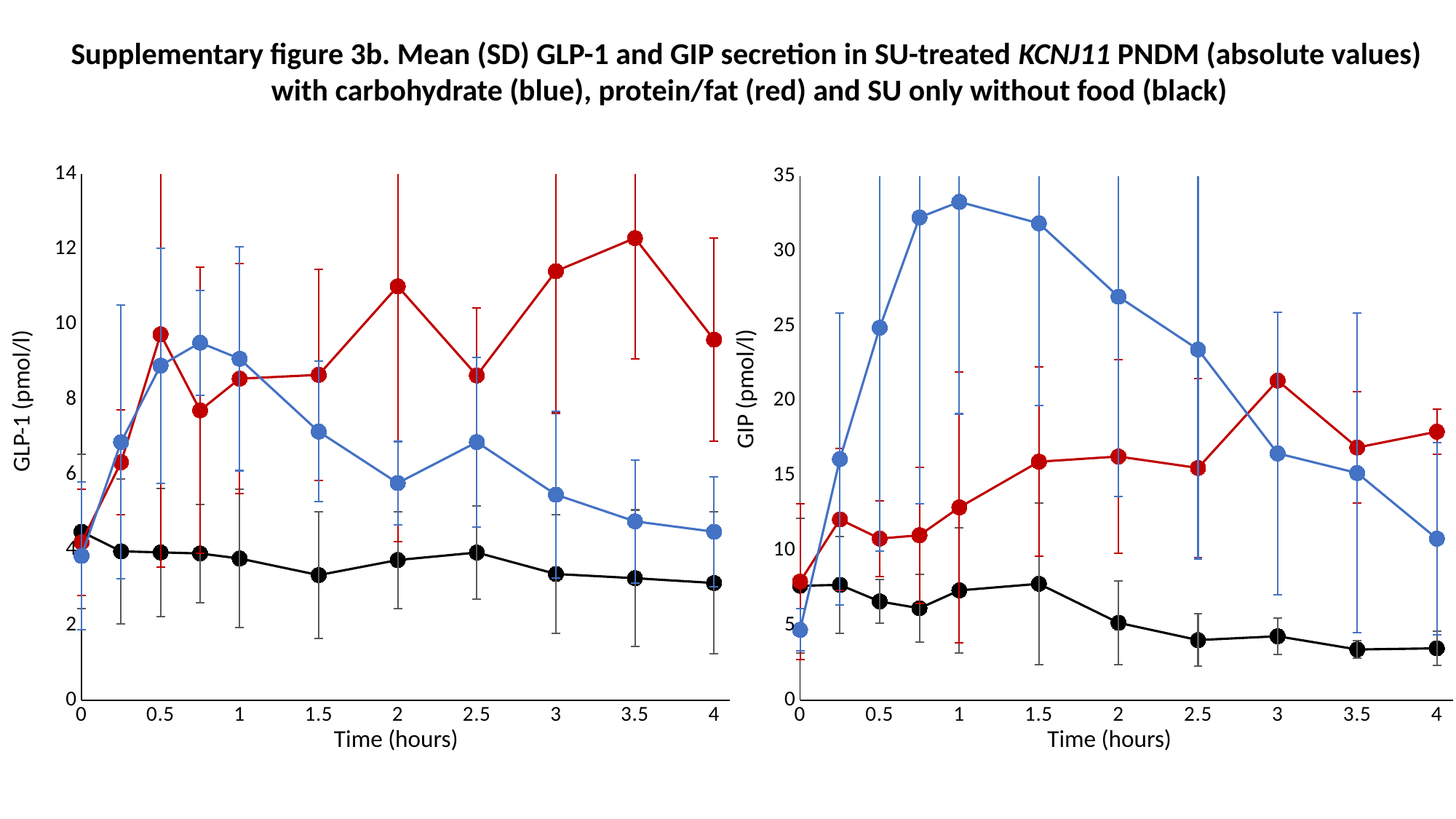

Supplementary figure 3b. Mean (SD) GLP-1 and GIP secretion in SU-treated KCNJ11 PNDM (absolute values)
with carbohydrate (blue), protein/fat (red) and SU only without food (black)
### Chart
| Category | | | |
|---|---|---|---|
### Chart
| Category | | | |
|---|---|---|---|GIP (pmol/l)
GLP-1 (pmol/l)
Time (hours)
Time (hours)

## Slide 7
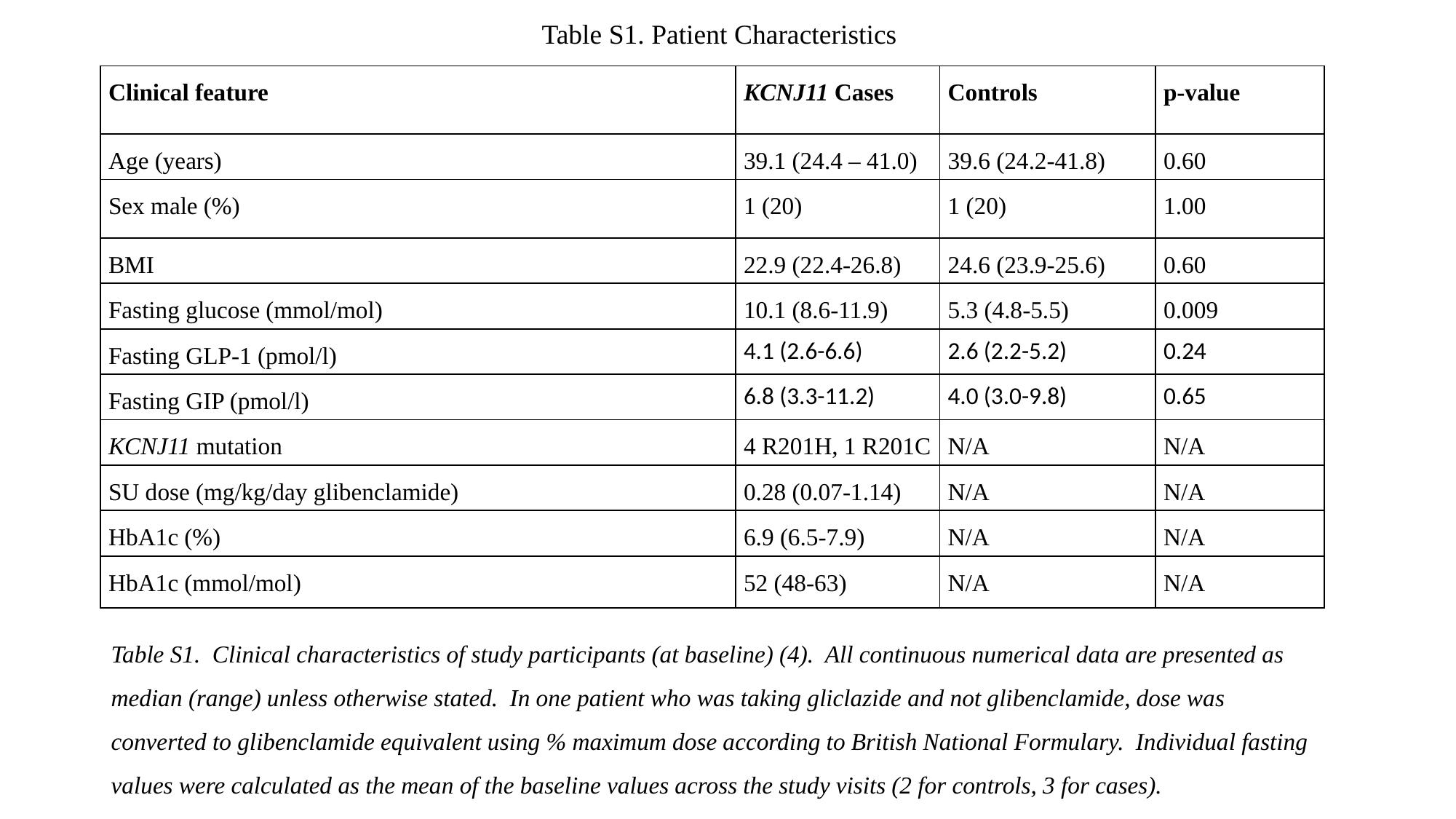

# Table S1. Patient Characteristics
| Clinical feature | KCNJ11 Cases | Controls | p-value |
| --- | --- | --- | --- |
| Age (years) | 39.1 (24.4 – 41.0) | 39.6 (24.2-41.8) | 0.60 |
| Sex male (%) | 1 (20) | 1 (20) | 1.00 |
| BMI | 22.9 (22.4-26.8) | 24.6 (23.9-25.6) | 0.60 |
| Fasting glucose (mmol/mol) | 10.1 (8.6-11.9) | 5.3 (4.8-5.5) | 0.009 |
| Fasting GLP-1 (pmol/l) | 4.1 (2.6-6.6) | 2.6 (2.2-5.2) | 0.24 |
| Fasting GIP (pmol/l) | 6.8 (3.3-11.2) | 4.0 (3.0-9.8) | 0.65 |
| KCNJ11 mutation | 4 R201H, 1 R201C | N/A | N/A |
| SU dose (mg/kg/day glibenclamide) | 0.28 (0.07-1.14) | N/A | N/A |
| HbA1c (%) | 6.9 (6.5-7.9) | N/A | N/A |
| HbA1c (mmol/mol) | 52 (48-63) | N/A | N/A |
Table S1. Clinical characteristics of study participants (at baseline) (4). All continuous numerical data are presented as median (range) unless otherwise stated. In one patient who was taking gliclazide and not glibenclamide, dose was converted to glibenclamide equivalent using % maximum dose according to British National Formulary. Individual fasting values were calculated as the mean of the baseline values across the study visits (2 for controls, 3 for cases).

## Slide 8
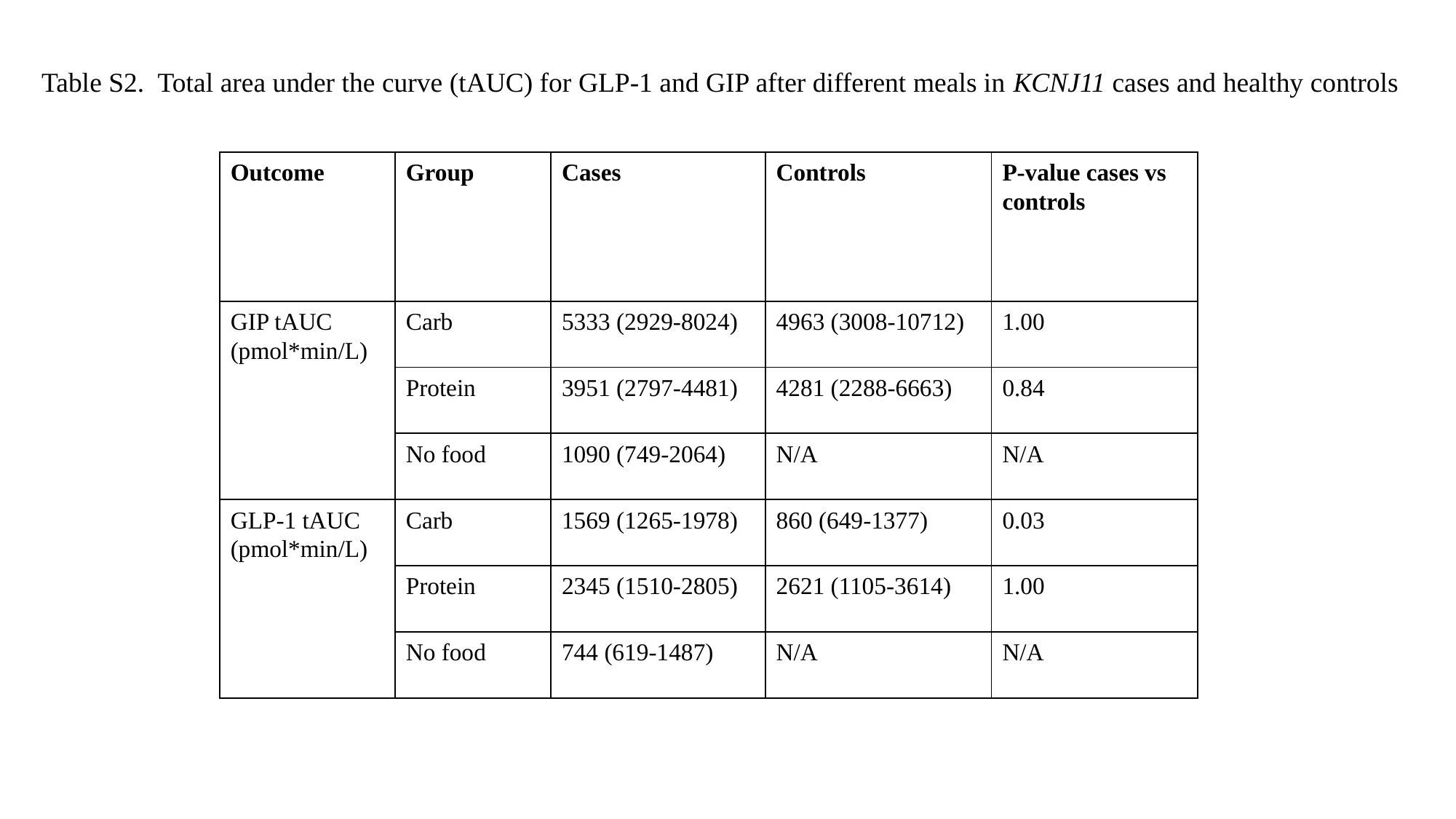

# Table S2. Total area under the curve (tAUC) for GLP-1 and GIP after different meals in KCNJ11 cases and healthy controls
| Outcome | Group | Cases | Controls | P-value cases vs controls |
| --- | --- | --- | --- | --- |
| GIP tAUC (pmol\*min/L) | Carb | 5333 (2929-8024) | 4963 (3008-10712) | 1.00 |
| | Protein | 3951 (2797-4481) | 4281 (2288-6663) | 0.84 |
| | No food | 1090 (749-2064) | N/A | N/A |
| GLP-1 tAUC (pmol\*min/L) | Carb | 1569 (1265-1978) | 860 (649-1377) | 0.03 |
| | Protein | 2345 (1510-2805) | 2621 (1105-3614) | 1.00 |
| | No food | 744 (619-1487) | N/A | N/A |
